# Development and validation of an individual alternative splicing prognostic signature in gastric cancer

**DOI:** 10.18632/aging.202507

**Published:** 2021-02-17

**Authors:** Shenghan Lou, Jian Zhang, Zhao Zhai, Xin Yin, Yimin Wang, Tianyi Fang, Yingwei Xue

**Affiliations:** 1 Department of Gastroenterological Surgery, Harbin Medical University Cancer Hospital, Harbin 150081, Heilongjiang Province, China; 2 Department of Thoracic Surgery, Harbin Medical University Cancer Hospital, Harbin 150081, Heilongjiang Province, China

**Keywords:** gastric cancer, alternative splicing, prognostic indicator

## Abstract

Gastric cancer (GC) is a heterogeneous disease with different clinical manifestations and prognoses. Alternative splicing (AS) is a determinant of gene expression and contributes to protein diversity from a rather limited gene transcript in metazoans. AS events are associated with different aspects of cancer biology, including cell proliferation, apoptosis, invasion, etc. Here, we present a comprehensive analysis of the prognostic AS profile in GC. GC-specific AS (GCAS) events were analyzed, and overall survival-associated GCAS (OS-GCAS) events were verified among the genome-wide AS events identified in The Cancer Genome Atlas (TCGA) database. In total, 1,287 GCAS events of 837 genes and 173 OS-GCAS events of 130 genes were identified. The parental genes of OS-GCAS events were significantly enriched in the development of GC. Protein-protein interaction (PPI) and OS-GCAS-associated splicing factor (SF) interaction networks were constructed. Multivariate Cox regression analysis with least absolute shrinkage and selection operator (LASSO) penalty was performed to establish a prognostic risk formula, representing 23 OS-GCAS events. The low-risk group had better OS than the high-risk group and lower immune and stromal scores. Cox proportional hazard regression was applied to generate an AS-clinical integrated prognostic model with a considerable area under the curve (AUC) value in both the training and validation datasets. Our study provides a profile of OS-GCAS events and an AS-clinical nomogram to predict the prognosis of GC.

## INTRODUCTION

Gastric cancer (GC) is the fifth most frequently diagnosed cancer and the third leading cause of cancer-related mortality worldwide, with an incidence of approximately 1,000,000 cases, accounting for approximately 800,000 deaths every year [1, 2]. Owing to the lack of early symptoms, most GC patients are diagnosed at an advanced stage [3–5]. Despite effective treatment, relapse and metastasis are common for advanced GC, which causes a quite low survival rate [3, 4, 6]. Currently, the tumor-node-metastasis (TNM) system is still the most common assessment system in predicting GC prognosis. However, the prognosis of GC patients within the same TNM stage is usually different, suggesting that the TNM system is not sufficiently satisfactory to predict GC prognosis [7–9]. Thus, a novel effective signature for predicting the prognosis of GC is urgently needed.

Alternative splicing (AS) is one of the most critical posttranscriptional regulatory processes and modifies more than 90% of all human genes [10]. The process of AS significantly contributed to protein diversity by generating different mRNA isoforms from a single gene [11]. Splicing abnormalities may cause a series of consequences from changing the stability to adding or deleting structural domains, modifying the interactive relationship of proteins, which significantly alters the abundance and complexity of the organism’s protein-protein interactions (PPIs) [12, 13]. Abnormal AS events participate in several tumorigenic processes, such as proliferation, apoptosis, hypoxia, angiogenesis, immune escape and metastasis [14, 15]. More importantly, growing evidence has demonstrated that AS has clinical potential in cancer therapy [12, 16, 17]. Thus, cancer-specific AS events might serve not only as prognostic signatures but also as potential therapeutic targets.

Despite the indispensable role of AS in oncogenesis, systematic analyses of the clinical significance of AS events in GC and the potential regulatory mechanism are still lacking. Thus, our study aimed to conduct systematic profiling of genome-wide AS events and their prognostic value associated with GC. First, we identified GC-specific AS (GCAS) events and investigated the relationship between GCAS events and overall survival (OS). The potential biological function and underlying molecular regulatory network of these OS-GCAS events were further explored. Second, we constructed an AS prognostic signature, which could stratify risk for patients with GC. We also uncovered the distinct clinical, molecular and immune features between high- and low-risk patients. Finally, by integrating the AS signature with clinical characteristics, an AS-clinical nomogram with high performance was established for clinical application.

## RESULTS

### Overview of AS events in the TCGA GC cohort

We preliminarily detected 119,697 AS events from 14,433 genes, which accounted for approximately 70% of protein-coding genes [18]. A large proportion of AS events could only be detected in a few samples, and specific splicing isoforms were barely detected (PSI value < 0.05). Then, we implemented a series of filters (percentage of samples with PSI values ≥ 75 and average PSI value ≥ 0.05) to obtain a reliable set of AS events in GC. Consequently, a total of 37,139 AS events were obtained from 10,380 genes, which were used for further analysis. Among these AS events, they were comprised of “15,816 ES events from 6,462 genes”, “6,633 AP events from 4,016 genes”, “5,988 AT events from 3,665 genes”, “3,285 AA events from 2,404 genes”, “2,691 AD events from 1,986 genes”, “2,564 RI events from 1,780 genes” and “162 ME events in 156 genes” (Supplementary Figure 1A). Among these seven types of splicing patterns, ES was the most frequent splicing pattern, followed by AP and AT, while ME was the least frequent splicing pattern. Of note, the number of AS events far exceeded the total number of genes, which indicated that one gene might undergo multiple splicing patterns. As shown in Supplementary Figure 1B, most genes contained two or more AS events, and several genes could have up to five different splicing modes.

### Identification of GC-associated AS events

**Figure 1:**
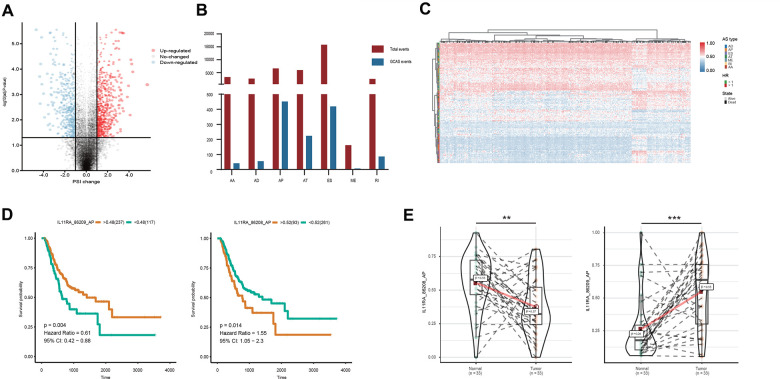
**Profiling of AS events identified in the TCGA GC cohort.** (**A**) GCAS events between GC and adjacent normal tissues were visualized in a volcano plot. (**B**) The number of GCAS events and total AS events were depicted according to the seven AS types. (**C**) Heat map for the PSI values of 173 OS-GCAS events identified in 354 GC patients. (**D**) Kaplan-Meier curves for the paired survival-related GCAS events (IL11RA_86208_AP and IL11RA_86209_AP). (**E**) Violin plots for the PSI values of the paired survival-related GCAS events (IL11RA_86208_AP and IL11RA_86209_AP) between GC and adjacent normal tissues.

To identify GC-associated AS events, we compared the PSI values between 33 paired tumor and normal tissues. According to the defined threshold, a total of 1,287 GCAS events were identified from 837 genes, which included 738 upregulated AS events from 625 genes and 549 downregulated AS events from 509 genes (Figure 1A and Supplementary Table 1). The proportion of AS splicing modes between the GCAS profiles and the entire AS profile was inconsistent (Figure 1B). A larger proportion of ES events (42.6%) were detected among all AS profiles than among GCAS events (32.6%). Moreover, AP events accounted for 17.9% of events among all AS profiles and accounted for 35.1% of GCAS events. Forty percent of the splicing-parent genes possessed more than one GCAS event. However, the proportion of splicing-parent genes derived from more than two GCAS events was very low (Supplementary Figure 1C).

### Identification of OS-GCAS events

To investigate the relationship between GCAS and OS in GC patients, we performed a univariate Cox regression analysis of 1,287 GCAS events in 354 patients. As shown in Figure 1C, 173 OS-GCAS events were identified from 130 genes (Supplementary Table 2). The proportion of AS splicing modes between the OS-GCAS and GCAS profiles was also inconsistent (Supplementary Figure 1D). The AP pattern (69 cases) contained the most OS-GCAS events, followed by the ES pattern (55 cases) and AT pattern (29 cases), while the AA pattern (2 cases) contained the fewest OS-GCAS events. Although 33.3% of splicing-parent genes had two or more OS-GCAS events, almost all the genes had only one splicing pattern (Supplementary Figure 1E). Of note, among the 130 splicing-parent genes, 38 genes contained paired survival-related GCAS events. For example, the AP of IL11RA in exon site 1 (IL11RA_86209_AP) was a favorable prognostic indicator, whereas the AP of IL11RA in exon site 2 (IL11RA_86208_AP) was a poor prognostic indicator (Figure 1D). Interestingly, the splicing pattern of all the paired survival-related GCAS events was either AP or AT, and the expression of the paired survival-related GCAS events was opposite between tumor and normal tissues (Figure 1E).

### Enrichment and interaction analysis of OS-GCAS events

**Figure 2:**
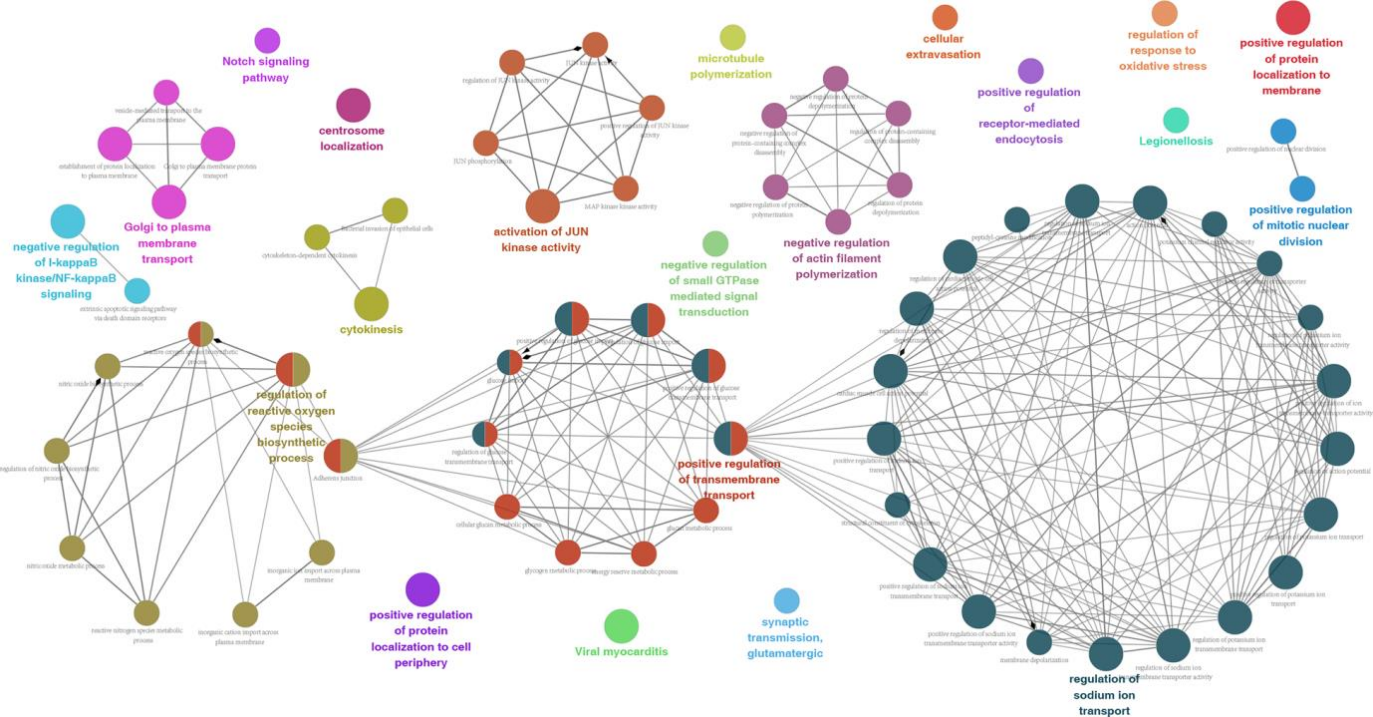
**Potential biological functions of 173 OS-GCAS events in GC.**

All 130 splicing-parent genes derived from 173 OS-GCAS events were assessed by functional and pathway enrichment analysis to explore the underlying mechanisms of the OS-GCAS events. The results revealed that genes were enriched in GO categories closely related to GC development, such as “microtubule polymerization”, “centrosome localization” and “cytokinesis” (Figure 2 and Supplementary Table 3). In addition, some canonical pathways involved in GC metastasis and recurrence were also enriched, such as the “Notch signaling pathway”, “adherens junction” and “bacterial invasion of epithelial cells” (Figure 2 and Supplementary Table 3).

Since AS events could inevitably affect protein characteristics, it is necessary to investigate these OS-GCAS events at the protein level [19]. A protein interaction network of OS-GCAS events was constructed not only to provide an overview of the interactions under normal conditions but also to reveal the potential influence of OS-GCAS events on the entire network. After removing the isolated nodes, 79 genes were mapped in the protein interaction network, and these parental genes were significantly correlated with each other (Supplementary Figure 2A). The top three hub genes were SORBS1, BPTF and SEPT2, which were determined by the maximal clique centrality method. The MCODE algorithm identified three individual modules from the whole protein interaction network (Supplementary Figure 2B–2D).

### The network of OS-GCAS events and SFs

**Figure 3:**
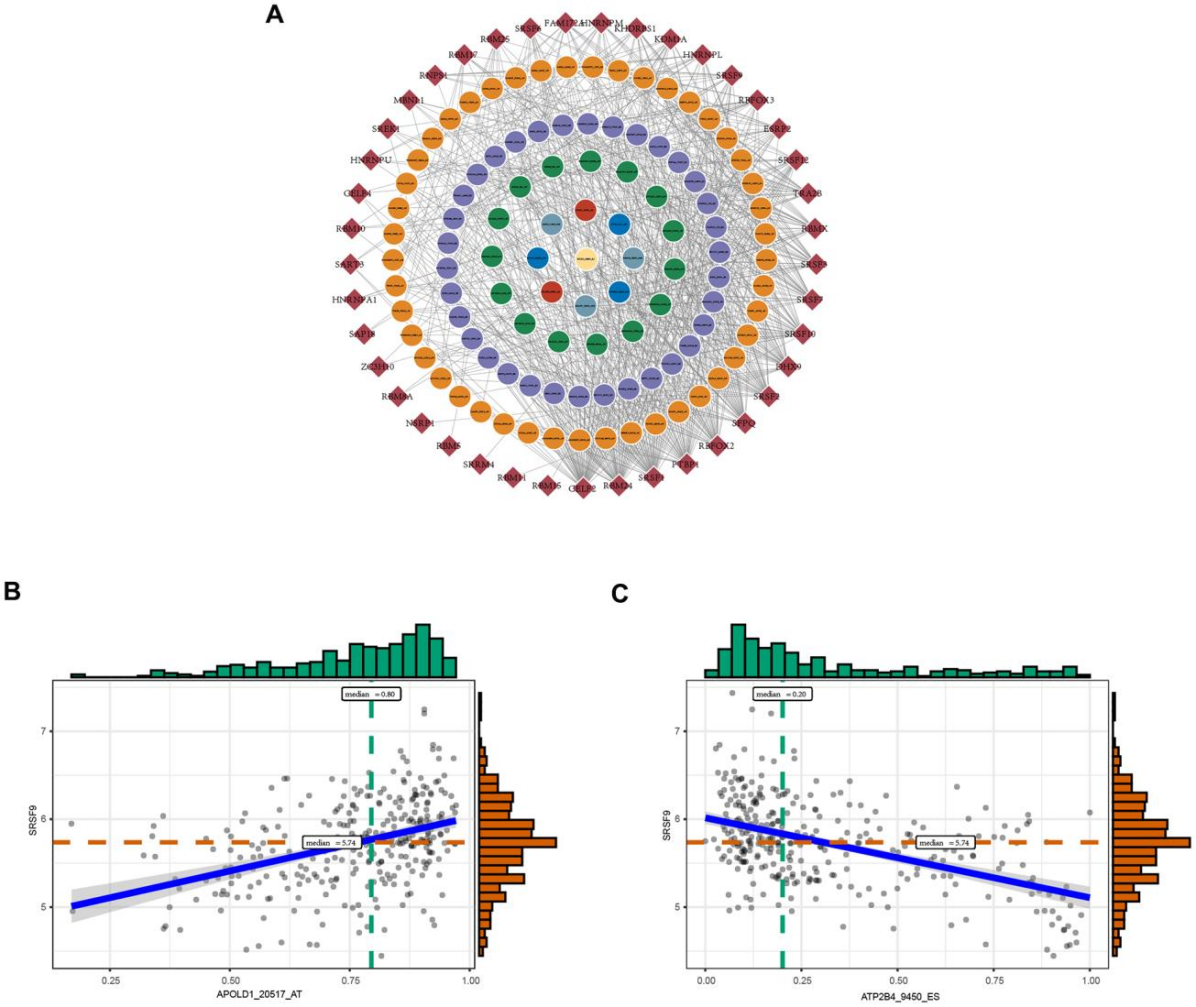
**The regulatory splicing correlation network in GC.** (**A**) The correlation of OS-GCAS events with SFs is shown in network plots. The circular node represents the OS-GCAS event. The diamond node represents the SF. (**B**) Representative positive correlations between OS-GCAS events and SFs are shown in scatter plots. (**C**) Representative negative correlations between OS-GCAS events and SFs are shown in scatter plots.

SFs are critical regulators of AS events that bind to pre-mRNAs and influence exon selection and splicing site choice [20]. It has been determined that dysregulated AS events within the tumor microenvironment may be regulated by a limited number of SFs [21]. Thus, correlation analysis was performed to explore the potential interaction networks of OS-GCAS events and SFs. As shown in Figure 3A, 103 of 173 OS-GCAS events were significantly associated with 41 experimentally validated SFs in the regulatory network (|R| ≥ 0.4 and *P*-value < 0.05). Among the networks, most SFs were significantly correlated with more than one OS-GCAS event, while a single OS-GCAS could be regulated by up to 21 different SFs (Figure 3A). Moreover, we found that some SFs played opposite roles in the regulation of different AS events. For example, the expression of SRSF9 was negatively correlated with ATP2B4_9450_ES but positively correlated with APOLD1_20517_AT (Figure 3B).

### Construction and evaluation of the AS prognostic signature

**Figure 4:**
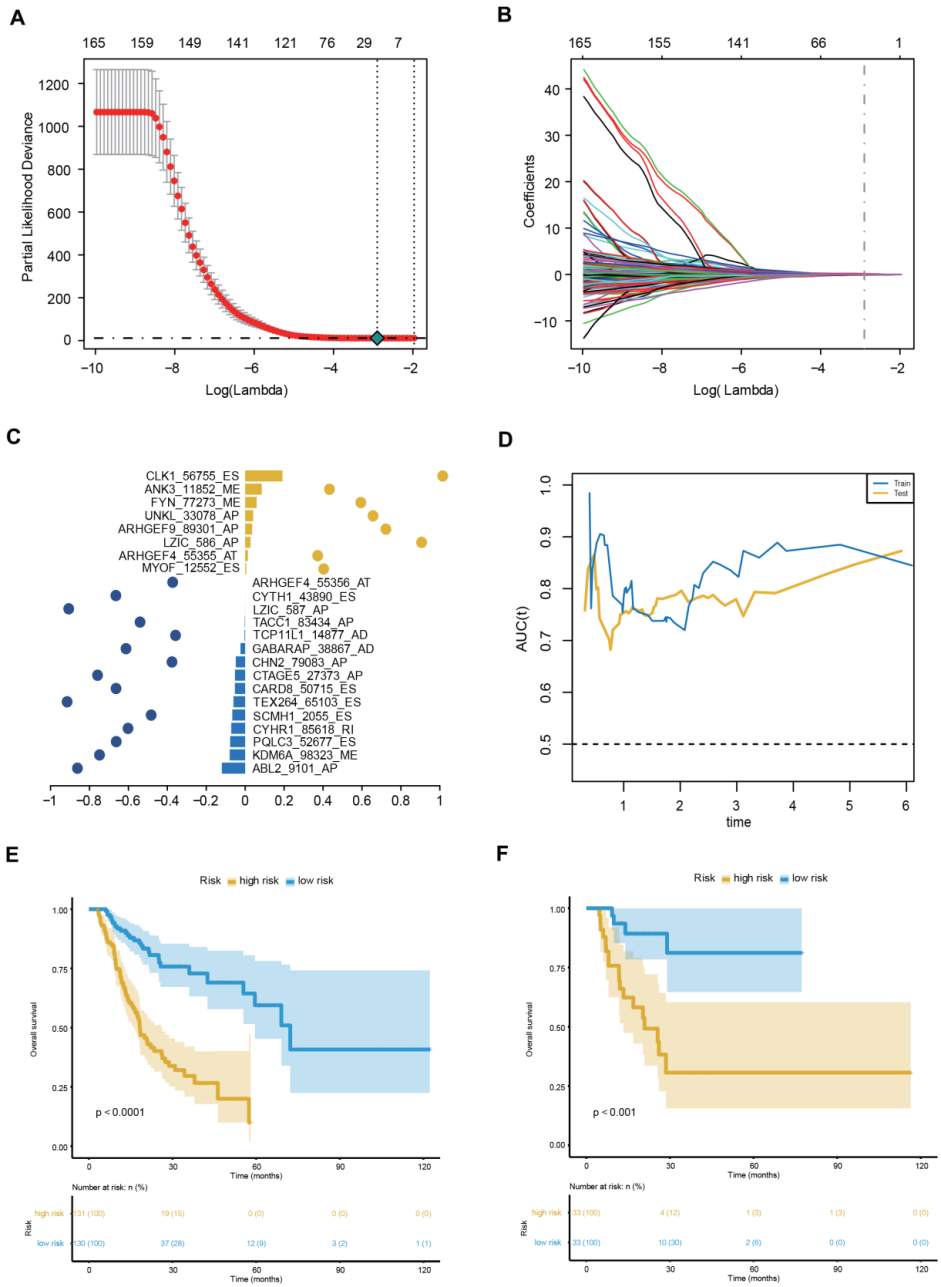
**Construction and evaluation of the 23-AS event prognostic signature for GC patients.** (**A**, **B**) LASSO regression analysis of OS- GCAS events. (**C**) The 23 OS-GCAS events included in the signature. Corresponding coefficients from multivariate Cox regression using LASSO and log10(HRs) are depicted by horizontal bars and dots, respectively. (**D**) Time-dependent ROC curves for the 23-AS event signature in the training and validation datasets. (**E**, **F**) Kaplan-Meier curves with difference detection by log-rank test for patients from the training and validation datasets.

To facilitate the application of AS events in the clinical monitoring of the prognosis of GC patients, we applied a LASSO penalty with a multivariate Cox regression analysis to the 173 OS-GCAS events in the training set of 261 patients. A total of 23 features were identified with nonzero coefficients (Figure 4A, 4B). These 23 LASSO-selected features were used to build the AS prognostic signature. The coefficients and PSI values of these 23 OS-GCAS events were used together to calculate the risk scores for both the training and validation datasets (Figure 4C).

This 23-AS event prognostic signature showed excellent performance in both datasets (Figure 4D). The C-index values of the signature were 0.725 and 0.762 in the training and validation datasets, respectively. The signature significantly stratified patients into low- and high-risk groups in the training set and validation set (Figure 4E, 4F). Significant RMS time ratios were also observed in the two datasets (Table 1). Moreover, the 23-AS event prognostic signature remained an independent prognostic factor in multivariate analyses after adjusting for clinical and pathologic factors, such as age, TNM stage and microsatellite instability (MSI) status (Table 2).

**Table 1:** Restricted mean survival (RMS) time ratio between the two risk groups in different datasets.

**Dataset**	**N_high-risk_**	**N_low-risk_**	**RMS_HRisk_**	**RMS_LRisk_**	**RMS ratio**	***P*-value**
			**(95% CI) ^a^**	**(95% CI) ^a^**	**(95% CI) ^b^**	
Training dataset	130	131	25.8 (21.8-29.9)	46.4 (42.5-50.3)	0.56 (0.47-0.67)	<0.001
Validation dataset	33	33	34.3 (22.1-46.5)	66.1 (55.9-76.3)	0.52 (0.35-0.77)	<0.001

^a^RMS time: months.

^b^RMS ratio = RMS_HRisk_/RMS_LRisk_.

**Table 2:** Multivariate Cox proportional hazard regression in the training and validation datasets.

	**Training dataset (n =261)**						**Validation dataset (n = 66)**					
	**Univariate regression**			**Multiple regression**			**Univariate regression**			**Multiple regression**		
	**HR**	**95% CI**	***P*-value**	**HR**	**95% CI**	***P*-value**	**HR**	**95% CI**	***P*-value**	**HR**	**95% CI**	***P*-value**
Age	***1.02***	***(1.003, 1.037)***	***0.022***	***1.025***	***(1.006, 1.043)***	***0.008***	1.005	(0.958, 1.055)	0.825			
Gender												
Female	Reference						Reference					
Male	1.186	(0.810, 1.736)	0.38				2.39	(0.808, 7.069)	0.115			
MSI status												
MSS	Reference						Reference			Reference		
MSI-H	0.685	(0.421, 1.114)	0.127				0.709	(0.200, 2.519)	0.595	2.28	(0.553, 9.409)	0.254
MSI-L	1.145	(0.681, 1.925)	0.611				***4.436***	***(1.594, 12.343)***	***0.004***	2.786	(0.973, 7.976)	0.056
TNM stage												
Stage I	Reference			Reference			Reference			Reference		
Stage II	1.845	(0.843, 4.036)	0.125	1.543	(0.697, 3.413)	0.284	1.018	(0.170, 6.099)	0.985	1.214	(0.196, 7.500)	0.835
Stage III	***2.902***	***(1.389, 6.062)***	***0.005***	***2.222***	***(1.055, 4.682)***	***0.036***	3.927	(0.883, 17.464)	0.072	2.919	(0.621, 13.713)	0.175
Stage IV	***4.128***	***(1.793, 9.501)***	***0.001***	***3.737***	***(1.598, 8.739)***	***0.002***	***7.888***	***(1.312, 47.411)***	***0.024***	4.385	(0.612, 31.440)	0.141
Molecular subtype												
CIN	Reference						Reference					
EBV	0.718	(0.347, 1.487)	0.372				1.483	(0.319, 6.901)	0.616			
GS	0.968	(0.573, 1.635)	0.903				2.453	(0.912, 6.599)	0.075			
HM-SNV	0.974	(0.239, 3.974)	0.971				2.277	(0.280, 18.492)	0.441			
MSI	0.618	(0.374, 1.021)	0.06				0.783	(0.210, 2.927)	0.717			
Neoplasm grade												
G1	Reference						NA					
G2	0.56	(0.173, 1.809)	0.333				Reference					
G3	0.754	(0.238, 2.394)	0.632				2.583	(0.949, 7.029)	0.063			
Risk score	***8.182***	***(5.357, 12.496)***	***< 0.001***	***7.765***	***(4.834, 12.475)***	***< 0.001***	***19.3***	***(6.015, 61.925)***	***< 0.001***	***13.545***	***(3.295, 55.687)***	***< 0.001***

Furthermore, we performed sensitivity analyses according to age, sex and TNM stage to evaluate the robustness of the 23-AS event prognostic signature in different clinical subgroups. The prognostic signature could stratify patients into significantly different prognostic groups in all the subgroups, which indicated that this prognostic signature might predict OS independently of clinical characteristics in GC (Supplementary Figure 3).

### Clinical, molecular and immune features underlying the 23-AS event prognostic signature

**Figure 5:**
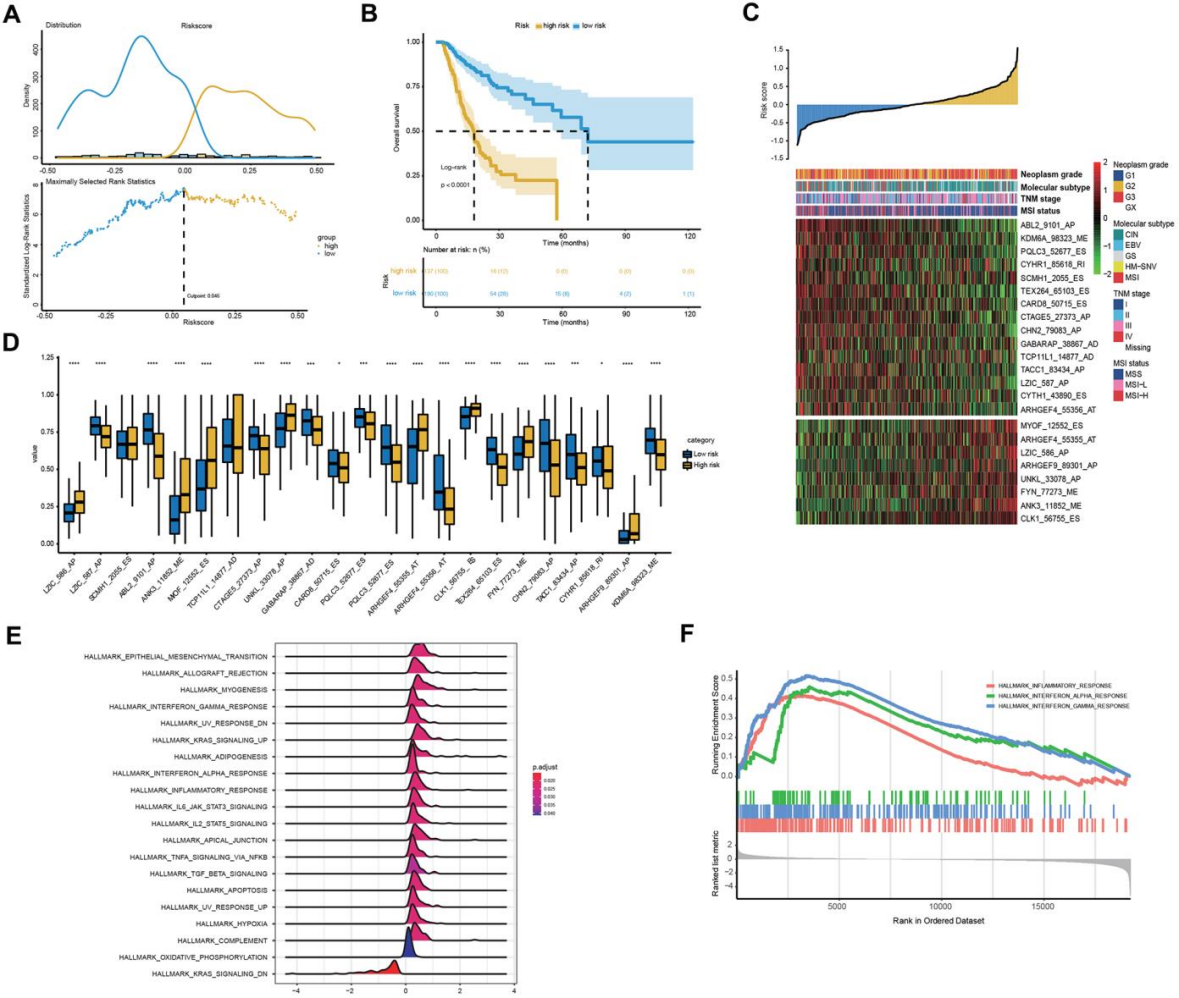
**Clinical and molecular features underlying the 23-AS event signature.** (**A**) The optimal cut-off of the 23-AS event signature in the entire TCGA dataset. (**B**) Kaplan-Meier curve with difference detection by log-rank test for patients in the entire TCGA dataset. (**C**) Heat map for the expression patterns of the 23 OS-GCAS events for the entire TCGA sample set sorted by the risk score in ascending order. (**D**) The differential expression of the 23 OS-GCAS events between the high- and low-risk groups. (**E**, **F**) GSEA of the 50 hallmark gene sets between the high- and low-risk groups.

To explore the relationship between patient characteristics and the 23-AS event prognostic signatures, patients in the entire TCGA dataset were stratified into high- and low-risk groups according to the optimal cut-off of 0.046 (Figure 5A). Among the 327 patients, 137 patients were assigned to the high-risk group, and 190 patients were assigned to the low-risk group. The low-risk group had a significantly more favorable prognosis of OS than the high-risk group (Figure 5B). We found that almost all 23 LASSO-selected features were significantly differentially expressed between the two risk groups (Figure 5C, 5D). Moreover, the distribution of MSI status, TNM stage and molecular subtype were significantly different between the high- and low-risk groups (Figure 5C and Table 3).

**Table 3:** Differences in patient characteristics between the high- and low-risk groups in the entire TCGA dataset.

	**Total sample**	**High-risk group**	**Low-risk group**	***P*-value**
**MSI status**				***< 0.001***
MSS	221	107 (48.42%)	114 (51.58%)	
MSI-H	62	11 (17.74%)	51 (82.26%)	
MSI-L	44	19 (43.18%)	25 (56.82%)	
**TNM stage**				***0.006***
Stage I	44	10 (22.73%)	34 (77.27%)	
Stage II	108	41 (37.96%)	67 (62.04%)	
Stage III	140	62 (44.29%)	78 (55.71%)	
Stage IV	29	18 (62.07%)	11 (37.93%)	
**Molecular subtype**				***< 0.001***
CIN	194	92 (47.42%)	102 (52.58%)	
EBV	24	8 (33.33%)	16 (66.67%)	
GS	42	23 (54.76%)	19 (45.24%)	
HM-SNV	7	3 (42.86%)	4 (57.14%)	
MSI	60	11 (18.33%)	49 (81.67%)	
**Neoplasm grade**				0.11
G1	6	4 (66.67%)	2 (33.33%)	
G2	124	44 (35.48%)	80 (64.52%)	
G3	190	86 (45.26%)	104 (54.74%)	

We then performed GSEA to verify the difference in biological function and pathway between the high- and low-risk groups. The results revealed that some well-known pathways related to GC development, such as “apoptosis”, “hypoxia” and “epithelial-mesenchymal transition”, were significantly enriched in the high-risk group (Figure 5E). Intriguingly, immune-related pathways, such as “inflammatory response”, “interferon-alpha response” and “interferon-gamma response”, were also significantly enriched in the high-risk group (Figure 5F).

**Figure 6:**
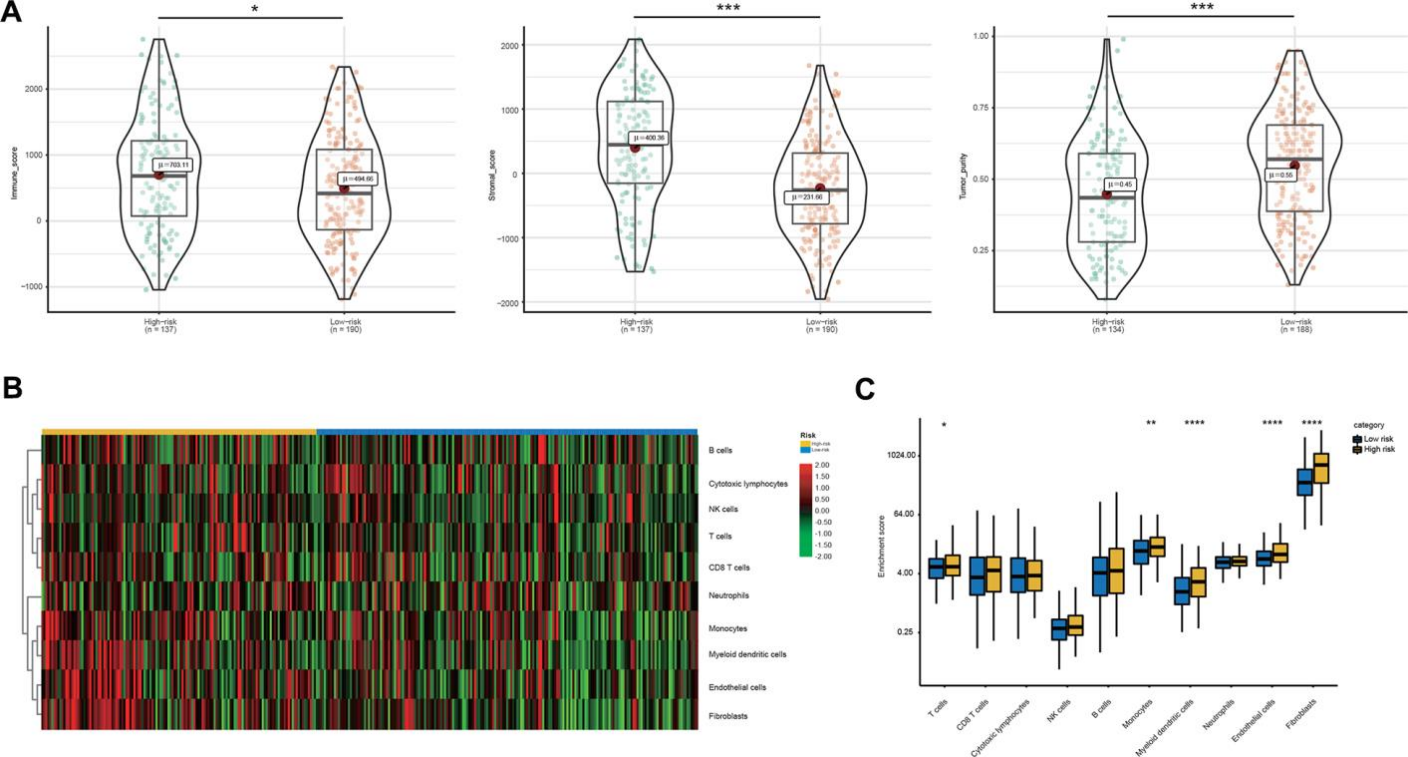
**Immune microenvironment features underlying the 23-AS event signature.** (**A**) Violin plots for the immune scores, stromal scores, and tumor purity between the high- and low-risk groups. (**B**) Heat map for immune cell infiltration between the high- and low-risk groups. (**C**) The differential expression of immune and stromal cells between the high- and low-risk groups.

Thus, we compared the tumor immune micro-environment between the high- and low-risk groups to comprehensively characterize their different immune features. Both the immune and stromal scores were significantly higher in the high-risk group (Figure 6A). Correspondingly, a lower tumor purity was also found in the high-risk group (Figure 6A). In detail, our studies of immune cell infiltration revealed that many types of immune cells were not randomly distributed across the two groups (Figure 6B, 6C). We found a significantly higher proportion of T cells, monocytes, myeloid dendritic cells, endothelial cells and fibroblasts in the high-risk group (Figure 6B, 6C). However, the proportions of the other five types of immune cells were comparable between the two groups (Figure 6B, 6C). These results indicated the activation of stromal and immune components in the tumor immune microenvironment of high-risk patients together with the activation of oncogenic pathways based on the proposed signatures, which likely contributed at least partially to the poor prognosis of these patients.

### Integrating the 23-AS event prognostic signature with clinical characteristics

**Figure 7:**
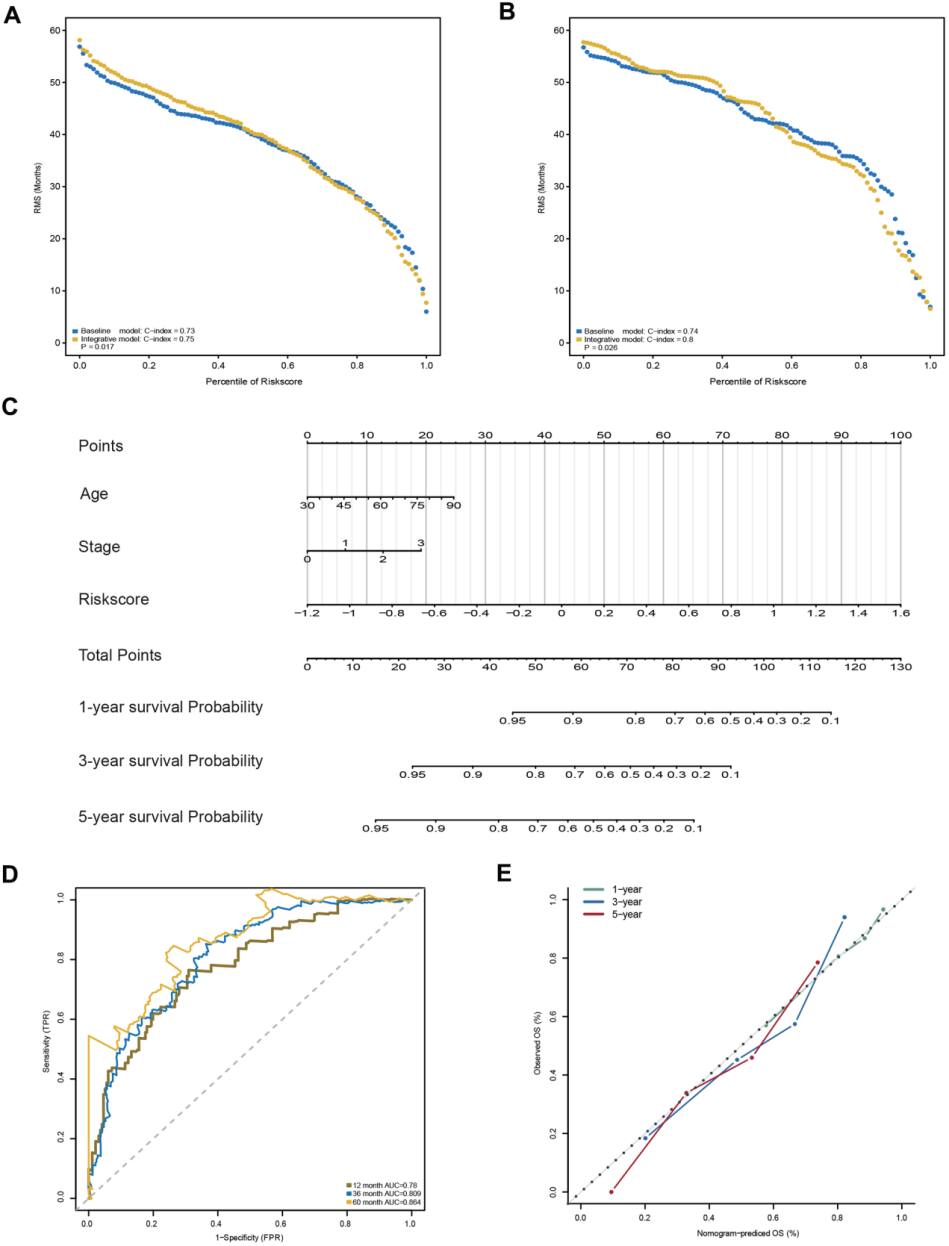
**Construction and evaluation of the AS-clinical nomogram for GC patients.** (**A**, **B**) RMS curves for the 23-AS event prognostic signature and the AS-clinical signature in the training and validation datasets. *P*-values represent the difference between the two signatures in terms of the C-index. (**C**) Nomogram prediction of 1-year, 3-year, and 5-year OS. For stage, 0 means TNM stage I, 1 means TNM stage II, 2 means TNM stage III, and 3 means TNM stage IV. (**D**) Time-dependent ROC curves for the nomogram at different time points. (**E**) Calibration curves of observed and predicted probabilities for the nomogram.

In addition to the 23-AS event prognostic signature, age and TNM stage were also determined as independent prognostic factors in the training dataset, which suggested their complementary value (Table 2). To further improve the prognostic accuracy, we integrated the 23-AS event prognostic signature with the other two independent prognostic factors, age and TNM stage, using the coefficients generated from the multivariate Cox regression model in the training dataset and derived an AS-clinical prognostic model as follows: Risk score = (1.948 × RSASS) + (0.348 × stage) + (0.022 × age). This integrated model was further validated in the validation dataset. Significant improvement in the assessment of survival was achieved with the AS-clinical model relative to the 23-AS event signature only (Figure 7A, 7B).

Moreover, a nomogram was established for model visualization and clinical application (Figure 7C). The ROC curve confirmed that the nomogram showed great performance in predicting the prognosis of GC (Figure 7D). The calibration curve also presented an optimal prediction for 1-year, 3-year and 5-year OS compared with the actual observations (Figure 7E). All these findings indicated that the nomogram built in our study might contribute significantly to the prediction of prognosis for patients with GC.

## DISCUSSION

AS is a critical posttranslational modification process that generates multiple mRNA and protein isoforms with distinct structural, regulatory and functional properties [11, 12]. It has been determined that abnormal AS events contribute to numerous diseases, including several types of cancers [14, 15]. In particular, accumulating evidence has proven that the specific dysregulation of AS events plays critical roles in GC initiation, progression and metastasis [22]. For instance, CD44, MUTYH, HOXA10 and MRPL33 splice variants participate in the carcinogenesis, proliferation, metastasis and drug resistance of GC [23–27]. However, previous studies mainly focused on monogenic isoforms with limited sample sizes, which lacked a general view of all AS events. Hence, we systematically profiled AS events in a large-scale GC cohort to characterize the role of AS events in the tumorigenesis and prognosis of GC.

In the current study, a total of 37,139 AS events from 10,380 genes were detected after the rigorous filter, which indicated that AS is a common process in GC. By comparing the tumor and paired normal tissues, 1,287 AS events from 837 genes were detected as GCAS events. As expected, all these experimentally validated splice variants were also identified by our procedure, suggesting that the GCAS events identified in our study are ubiquitous in GC. Interestingly, we found that GC shared some common cancer-specific AS events with colorectal and head-neck squamous cell cancers, which also illustrated the role of shared AS events in cancer tumorigenesis and development [28, 29].

Among the 1,287 detected GCAS events, 173 OS-GCAS events from 130 genes were identified by univariate Cox regression analysis. In our study, a higher proportion of AP and AT events was found in the prognostic AS profile, even though ES events are the predominant components in the entire AS profile and the cancer-specific AS profile (Supplementary Figure 1A, 1D). Previous studies also confirmed that AP and AT splicing types often confer splicing isoform-specific localization and control the survival and migration of cancer cells [30, 31]. Moreover, for OS-GCAS events of AP and AT splicing types, splicing isoforms at different splice sites had distinct expression patterns and prognostic values. All the results suggested that AP and AT splicing types played important roles in the development and progression of GC.

The potential biological function and underlying molecular regulatory network of these OS-GCAS events were further explored. We found that their splicing-parent genes were significantly enriched in GC initiation and maintenance by GO term and KEGG pathway enrichment analysis. In the PPI network, we found that 79 proteins interacted closely with each other. The complexity of the network indicated that the prognosis of GC was not driven by a single AS-relevant protein; it was a process regulated by the whole integrated system.

Given the influence of SFs on the process of RNA splicing, we performed an integrated analysis of SFs and OS-GCAS events to explore the underlying mechanism of the splicing pathway involved in GC patient survival [32]. The splicing correlation network showed distinguished interactions between SFs and OS-GCAS events. Of note, we found that a given SF might play dual roles in the positive and negative regulation of different AS events and that the same OS-GCAS event could be synergistically or antagonistically regulated by different SFs, which suggested that multiple SFs could affect the survival of GC patients by synergistically regulating the AS events of genes. The relationships between AS events and SFs might be suitable to consider as a dynamic interaction network instead of a simple “one-to-one” pattern.

To explore the prognostic significance of AS events, we constructed a robust signature for GC. The 23-AS event prognostic signature showed excellent performance in both the training and validation datasets. The 23-AS event prognostic signature precisely stratified risk. Patients in the high-risk group had a poorer prognosis than those in the low-risk group. Subgroup analysis also indicated that the prognostic signature was stable in different situations. All the results suggest that the 23-AS event prognostic signature could provide patients with new predictive information and facilitate patient assessment.

Of note, we found that the distribution of MSI status, TNM stage and molecular subtype was significantly different between the high- and low-risk groups, which indicated different biological functions between the high- and low-risk groups. Consistent with the results, GSEA revealed that several GC-associated pathways were significantly enriched in the high-risk group. Intriguingly, immune-related pathways were also significantly enriched, which suggested an enhanced immune phenotype in the high-risk group.

Correspondingly, we further estimated the population abundance of tissue-infiltrating immune and stromal cell populations to characterize the tumor immune microenvironment of the high- and low-risk groups. Generally, significantly higher immune and stromal scores were found in the high-risk group, which was characterized by a low tumor purity. Consistent with our study, previous studies have determined that low tumor purity is associated with poor prognosis and an enhanced immune phenotype [33–35]. Specifically, significantly higher proportions of T cells, monocytes, myeloid dendritic cells, endothelial cells and fibroblasts were identified in the high-risk group. The higher proportion of T cells in the high-risk group might be an essential factor for the immunotherapy response [36].

To further improve the prognostic accuracy, we integrated the 23-AS event prognostic signature with clinicopathologic factors. The prediction performance of the integrative AS-clinical prognostic model was superior to that of the single 23-AS event prognostic signature. Finally, a nomogram was established for model visualization and clinical application. The nomogram also showed great performance in predicting the prognosis of GC. Thus, we propose that the AS-clinical nomogram could serve as an individualized, single-sample estimate of survival of GC and might be readily translated to clinical practice.

In summary, our study comprehensively investigated the prognostic value of genome-wide AS events in GC and explored their potential biological function and the underlying splicing pathways of these 173 OS-GCAS events. More importantly, we constructed a 23-AS event prognostic signature to classify the risk of GC patients and identified differential clinical, molecular and immune features underlying the 23-AS event prognostic signature. In addition, combining data concerning age, TNM stage and the 23-AS event prognostic signature, we constructed an AS-clinical nomogram to predict the survival of GC patients. These results provide fundamental information for understanding the roles of the AS process and indicate the potential clinical implications of AS events in GC.

## MATERIALS AND METHODS

### Data acquisition and curation process

Patients who met the following criteria were included in The Cancer Genome Atlas (TCGA) GC cohort: (1) histologically confirmed primary GC; (2) alternative RNA splicing data available; (3) detailed clinicopathological and follow-up information available, including sex, age, TNM stage, neoplasm grade, microsatellite status, molecular subtype, and OS status; and (4) an OS time of over 90 days; this latter criterion was applied to, avoid immortal time bias. The alternative RNA splicing data of the TCGA GC cohort were downloaded from the TCGASpliceSeq dataset [37]. Corresponding clinical information and RNA-seq data were downloaded from the Genomic Data Commons data portal using the "GDCRNATools" package [38].

Splicing events in the TCGASpliceSeq dataset were divided into seven categories: exon skip (ES), retained intron (RI), alternate promoter (AP), alternate terminator (AT), alternate donor site (AD), alternate acceptor site (AA), and mutually exclusive exons (ME). Each splicing event was quantified by the percent spliced in (PSI) value, which ranges from 0 to 1 and represents the ratio of normalized read counts to indicate the inclusion of a transcript element over the total normalized reads for that event [39]. To generate a reliable set of AS events, we implemented a series of stringent filters, which included "percentage of samples with a PSI value ≥ 75%" and "average PSI value ≥ 0.05" [28, 29]. The missing PSI values were further filled in using the k nearest neighbors (KNN) algorithm [40].

### Identification of GCAS events and OS-GCAS events

To identify GCAS events, we applied the Wilcoxon matched-pairs signed-rank test to compare the PSI values between tumor tissues and matched adjacent normal tissues. The *P*-value was adjusted by the Benjamini-Hochberg (BH) method. GCAS events were defined as a median PSI value that varied more than 0.1 between tumor tissues and matched adjacent normal tissues with a BH-adjusted *P*-value < 0.05 [30, 41].

To determine the survival-associated GCAS events, we performed univariate Cox proportional hazards regression analysis to estimate the PSI values of GCAS events with OS. GCAS events with a *P*-value < 0.05 in the univariate Cox regression analysis were selected as OS-GCAS events.

### Functional enrichment analysis and protein interaction network

To investigate the potential functions of OS-GCAS events, we subjected their parent genes to enrichment analyses of Gene Ontology (GO) terms and Kyoto Encyclopedia of Genes and Genomes (KEGG) pathways using the ClueGO plug-in of Cytoscape [42]. For the functional enrichment analysis, a BH-adjusted *P*-value < 0.05 was considered statistically significant.

To observe the PPIs among the OS-GCAS events, we mapped their corresponding genes to the Search Tool for the Retrieval of Interacting Genes/Proteins (STRING) database [43]. A minimum required interaction score of 0.4 was used to identify the protein interaction results in STRING, which were further visualized by Cytoscape [44]. In addition, hub genes and specific modules of the protein interaction network were identified by the CytoHubba and Molecular Complex Detection (MCODE) plug-ins of Cytoscape [45, 46]. The maximal clique centrality (MCC) algorithm of CytoHubba was used to predict the top three hub genes in the PPI network. The MCODE options were set as degree cutoff = 2, K-core = 2, and node score cutoff = 0.

### Splicing factors (SFs) and the splicing correlation network

A total of 78 genes that participated in the process of alternative RNA splicing (GO:0000380) were obtained from the Molecular Signatures Database (MSigDB) [47]. The read count values of these SFs were extracted from the RNA-seq data, normalized by the trimmed means of M (TMM) method and further transformed by "voom" [48, 49]. Differentially expressed SFs were identified through the "limma" package, and a BH-adjusted *P*-value < 0.05 was used as the significance threshold [50]. Spearman's correlation analysis was conducted to explore the underlying correlation between the expression of the SFs and the PSI values of the OS-GCAS events. The absolute value of the correlation coefficient ≥ 0.4 with a *P*-value < 0.05 was considered statistically significant. The splicing correlation network of SFs and the OS-GCAS events was visualized by Cytoscape [44].

### Identification of the AS prognostic signature

To enhance the robustness of the AS prognostic signature, the entire TCGA cohort of 327 samples was first randomly distributed into two datasets (7:3), namely, the training and validation datasets. Next, to avoid overfitting in the multivariate model, the least absolute shrinkage and selection operator (LASSO) penalty was applied in the training dataset to build an optimal prognostic signature with the minimum number of OS-GCAS events. Ten-fold cross-validation was conducted to determine the optimal value of the penalty parameter λ, which gives the minimum partial likelihood deviance. Finally, a set of OS-GCAS events and their nonzero coefficients were identified, which was used to build the AS prognostic signature. The time-dependent receiver operator characteristic (ROC) curve and concordance index (C-index) were applied to assess the performance of the prognosis model.

The risk score for the AS signature was calculated for each sample via a linear combination of the selected features, weighted by the corresponding coefficients. Patients were divided into high- and low-risk groups using the cohort-specific median risk score as the cut-off for each dataset. Kaplan-Meier survival analysis was performed to compare patient prognosis between the high- and low-risk groups. Furthermore, the restricted mean survival (RMS) time was also used to compare the prognostic differences between the two groups [51]. The higher the RMS value was, the greater the prognostic difference. A *P*-value < 0.05 was considered statistically significant.

### Association between different risk groups and clinical, molecular, and immune features

Patients in the entire TCGA cohort were divided into high-risk and low-risk groups according to the optimal cut-off value, which was determined by the maximally selected rank statistics test. For the difference between the two groups, clinical features (age, sex, TNM stage, neoplasm grade, and survival status), molecular features (microsatellite status and molecular subtype), and immune features (immune score, stromal score, tumor purity, and immune cell infiltration) were analyzed.

Tumor purity was measured by “ABSOLUTE” [52]; immune and stromal scores were calculated by “ESTIMATE” [53]; and immune cell infiltration was estimated with “MCPcounter” [54]. Kaplan-Meier curve with log-rank test was used for survival data; Mann-Whitney test was used for continuous data; chi-square test was used for categorical data. A *P*-value < 0.05 was considered statistically significant. In addition, to further explore the difference in biological functions and pathways between high-risk and low-risk groups, gene set enrichment analysis (GSEA) was performed using the “hallmark gene sets” downloaded from MSigDB [47, 55]. A BH-adjusted *P*-value < 0.05 was considered statistically significant.

### Development and verification of an AS-clinical nomogram

Based on the results derived from multivariate analyses, we integrated age, TNM stage, and AS prognostic signature to generate an AS-clinical prognostic model by applying Cox proportional hazard regression in the training dataset. The AS-clinical prognostic model was then applied to the validation dataset for further validation. Next, the prognostic value of the AS-clinical model was compared with that of the AS signature in terms of C-index, and the results were revealed by the RMS curve [18]. RMS represents the life expectancy at five years for patients with different risk scores. Finally, based on the AS-clinical prognostic model, a nomogram was developed to estimate the individual survival probability of patients. The ROC and calibration curves were used to assess the discrimination and calibration ability of the AS-clinical nomogram.

## Supplementary Material

Supplementary Figures

Supplementary Table 1

Supplementary Table 2

Supplementary Table 3
