# Supplementary material for: Development and validation of an individual alternative splicing prognostic signature in gastric cancer
Source: Aging (Albany NY). 2021 Feb 17;13(4):5824–44. doi: 10.18632/aging.202507 (PMC7950272; doi:10.18632/aging.202507)
Supplement: Supplementary Figures [file aging-13-202507-s001.pdf]

SUPPLEMENTARY FIGURES

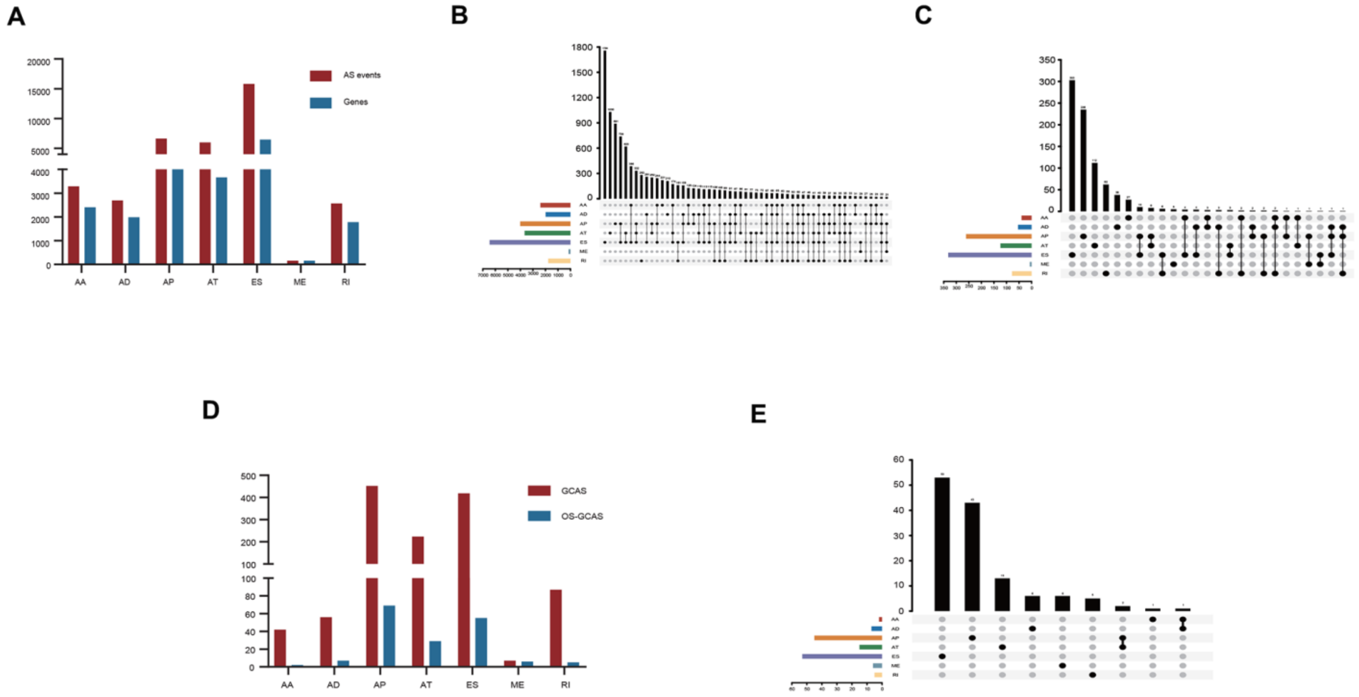

Supplementary Figure 1. Overview of AS event profiling in the TCGA cohort. (**A**) The numbers of AS events and corresponding genes are depicted according to the seven AS types. (**B**) UpSet plot for the intersection of the seven types of detected AS events. (**C**) UpSet plot for the intersection of the seven types of GCAS events. (**D**) The numbers of GCAS events and OS-GCAS events are depicted according to the seven AS types. (**E**) UpSet plot for the intersection of the seven types of OS-GCAS events.

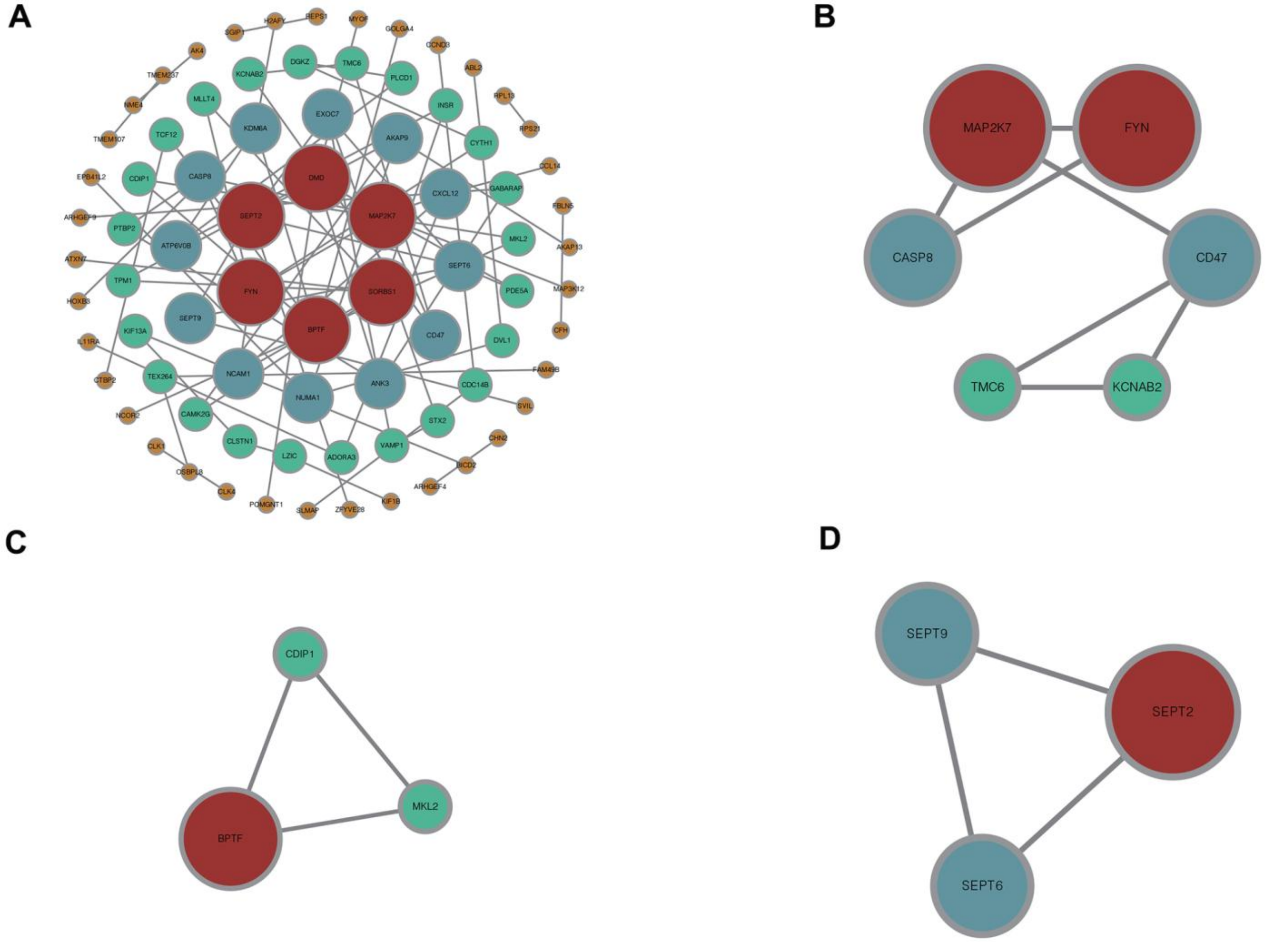

Supplementary Figure 2. PPI analysis of the 173 OS-GCAS events. (**A**) The PPI network for the interactome of 130 parental genes for 173 OS-GCAS events. (**B**–**D**) Three individual modules from the whole PPI network.

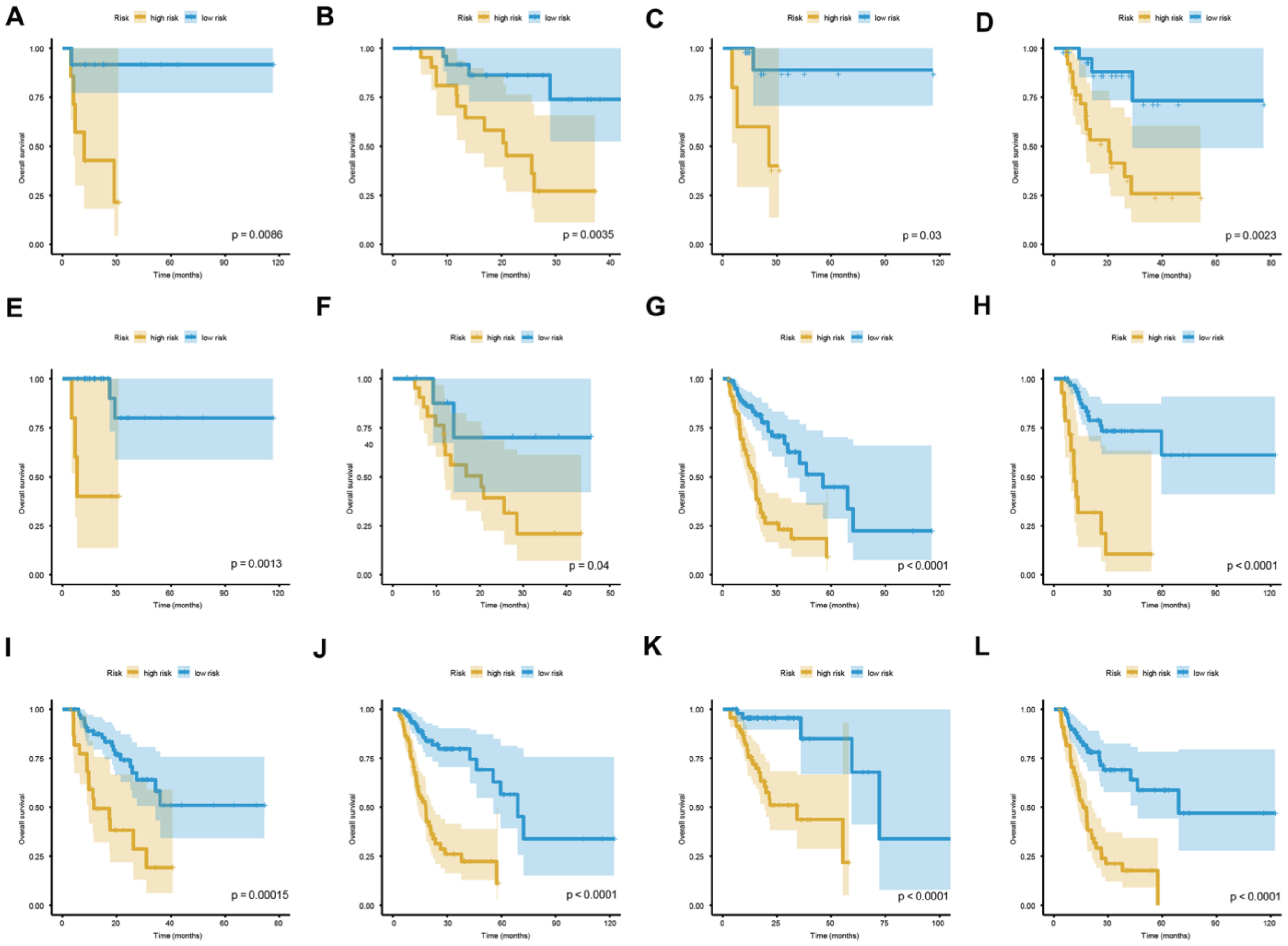

Supplementary Figure 3. Estimation of the prognostic value of the 23-AS event signature in diverse subgroups classified by different clinical characteristics. The dataset was classified into diverse subgroups according to age (< 60 years or ≥ 60 years), sex (female or male), and TNM stage (I + II or III + IV). (**A**–**F**) shows the validation dataset. (**G**–**L**) shows the training dataset.
